# Intimate Partner Violence and its associated factors among pregnant women receiving antenatal care. A Bayesian analysis approach

**DOI:** 10.1371/journal.pone.0304498

**Published:** 2024-07-11

**Authors:** Setognal Birara Aychiluhm, Kusse Urmale Mare, Kedir Y. Ahmed, Mahlet Seifu Demissie, Abay Woday Tadesse

**Affiliations:** 1 Department of Epidemiology and Biostatistics, Institute of Public Health, College of Medicine and Health Sciences, University of Gondar, Gondar, Ethiopia; 2 Rural Health Research Institute, Charles Sturt University, Orange, NSW, Australia; 3 Department of Nursing, College of Medicine and Health Sciences, Samara University, Samara, Ethiopia; 4 Translational Health Research Institute, Western Sydney University, Campbelltown, NSW, Australia; 5 Department of Radiology, School of Medicine, College of Medicine and Health Sciences, University of Gondar, Gondar, Ethiopia; 6 Curtin School of Population Health, Curtin University, Bentley, WA, Australia; 7 Dream Science and Technology College, Dessie, Amhara Region, Ethiopia; Jahangirnagar University, BANGLADESH

## Abstract

**Background:**

Intimate Partner Violence (IPV) is a major public health problem worldwide. In developing nations, including Ethiopia, the problem is under-reported and under-estimated. Therefore, this study attempts to assess intimate partner violence and its associated factors among pregnant women receiving antenatal care at public hospitals in Amhara region, Ethiopia.

**Methods:**

A health facility-based cross-sectional study design was employed. A sample of 418 pregnant women was selected using random sampling technique from 1^st^ May to 1^st^ June 2021. IPV was measured using the World Health Organization (WHO) Multi-country study of violence against women assessment tool. Data were entered into Epi-data 3.1 and exported into Stata 17 for further analysis. A Bayesian multivariable logistic regression analysis was carried out from the posterior distribution, and an adjusted odds ratio (AOR) with a 95% credible interval (CrI) was used to declare statistically significant variables.

**Results:**

The prevalence of any IPV among pregnant women was 31.3% [95% CrI 26.6%, 36.1%]. After adjusting a range of covariates, IPV during pregnancy was more likely among women whose husbands used substances [AOR = 4.33: 95% CrI 1.68, 8.95] and household decisions made by husbands only [AOR = 6.45: 95% CI 3.01, 12.64]. Conversely, pregnant women who attended primary [AOR = 0.47: 95% CrI 0.24, 0.81] and secondary [AOR = 0.64: 95% CrI 0.41, 0.92] educational levels, women who had four or more ANC visits antenatal care visits [AOR = 0.43: 95% CrI 0.25, 0.68], and women with no prior history of adverse birth outcomes [AOR = 0.48: 95% CI 0.27, 0.80] were less likely to experience IPV during pregnancy.

**Conclusion:**

The study revealed a relatively high prevalence of any IPV among pregnant women, with factors such as substance use by husbands and limited decision-making autonomy associated with increased IPV likelihood. Conversely, women with higher education levels, four and above antenatal care attendance, and no history of adverse birth outcomes showed a reduced likelihood of experiencing IPV during pregnancy. Therefore, targeted interventions to address substance use, empower women in decision-making, and promote education and healthcare access to mitigate IPV risk during pregnancy are recommended.

## Introduction

Intimate Partner Violence (IPV) is a public health problem and human rights violation, defined by the World Health Organization (WHO) as a physical, sexual, or psychological coercive act by a current or former partner or spouse to a woman [1]. The global prevalence of any IPV among all ever-partnered women varies from nation to nation [2] and 37% to 80% had happened in African, and South-East Asia Regions [3, 4]. In Ethiopia, the prevalence of IPV varies significantly across different regions, ranging from 25.4%-30% in the Amhara region [5, 6], and nearly 40% in the eastern [7] part of the country.

Prior studies conducted in low-income countries, including Ethiopia, have identified a wide range of factors associated with IPV during pregnancy, including partner alcohol consumption, education level of the women, age at first marriage, sociocultural factors, and socioeconomic factors [5–11]. Exposure to IPV during pregnancy has been linked to adverse maternal health outcomes such as various psychological, socioeconomic, and medical problems for affected women and a wide range of adverse birth outcomes, including miscarriages, low birth weight, premature birth, and small-for-gestational age [4, 11–15].

Efforts to prevent and control violence against women have included global, regional, and country-level strategies, legislative laws, and programs [8, 16–20]. Ethiopia, as a signatory to various international and regional human rights treaties, including the Convention on the Elimination of Discrimination against Women (CEDAW), recognizes the crucial role played by civil society organizations in advancing and safeguarding women’s rights [21–25]. Despite these recognitions and efforts, the prevalence of violence against pregnant women remains high in Ethiopia, persisting into the twenty-first century.

The existing research on intimate partner violence (IPV) has primarily relied on observed data, leading to estimations with greater standard errors. Recognizing the limitations of this approach, the current study employs Bayesian logistic regression, a robust statistical method known for reducing standard errors and enhancing parameter estimation stability [26, 27]. This choice is justified by the need to address gaps in previous research methodologies and aims to determine the prevalence of IPV and its associated factors among pregnant women. The Bayesian analysis approach utilized in this study is a powerful statistical method in health sciences research, emphasizing its potential to contribute valuable insights based on both existing data and prior information [28, 29]. Therefore, this study aimed to determine the prevalence of IPV and its associated factors among pregnant women using a powerful statistical approach in health sciences research that accounts both observed data at hand and prior knowledge.

## Methods and materials

### Study setting and participants

The study was conducted in randomly selected four public hospitals located in Amhara regional state, Namely Debre Birhan, Dessie, Woldia, and Bahir Dar Felege Hiwot Referral Hospitals, from May 1st to June 1st, 2021. Ethiopia’s Amhara Region is situated between 8°45’ and 13°45’ North latitude and 36°20’ and 40°20’ East longitude. Around 170000 square kilometers are thought to be the total land extent. Amhara is bordered to the north by the Tigray Region, to the east by Afar, to the south by Oromiya, to the southwest by Benishangul-Gumiz, and the west by Sudan. The world-famous Nile River and its source, Lake Tana, as well as historical sites like Gonder and Lalibela, are in this region. The historic Amhara region contains much of the highland plateaus above 1500 meters with rugged formations, gorges, and valleys, as well as millions of settlements for Amhara villages surrounded by subsistence farms and grazing fields.

In Amhara Regional State, there are 17 hospitals, 847 health centers, and 3,342 health posts [30]. All the pregnant women who visited the randomly selected hospitals for Antenatal care services during the study period were included in this study. However, women who were seriously ill during the study period were not included in this study.

### Sample size determination and sampling procedure

The sample size was calculated by using single population proportions formula with assumptions of a 95% confidence interval, 5% margin of error, 10% non-response rate, and using a 44.5% prevalence of any IPV among pregnant women, which was taken from a study conducted in western Ethiopia to get maximum sample size [31]. Then, the final calculated sample size becomes 418.


$$n=\frac{{\left(Za/2\right)}^{2}(P)(1-P)}{d^{2}}$$

Where, n = sample size, Zα/2 = critical value for normal distribution at 95% confidence level (1.96), p = prevalence of IPV during pregnancy, and d = marginal error.

From the seventeen hospitals found in the Amhara region, four public hospitals namely Debre Birhan, Dessie, Woldia, and Bahir Dar Felege Hiwot, were randomly selected by lottery methods using names and lists of the hospitals as sampling frame. Then, the calculated sample size was proportionally allocated to randomly select public hospitals based on their ANC visit flows. The arrival of pregnant mothers to the antenatal care clinic was assumed at random and the data collectors have interviewed each eligible pregnant woman until proportionally allocated sample size in each hospital is achieved.

### Study variables

#### Dependent variable

Any Intimate Partner Violence that encompasses women who experienced at least one of the three constructs of IPV (physical or emotional or sexual violence) during pregnancy were classified as any IPV experiences during the current pregnancy.

#### Independent variable

A range of maternal and paternal factors, which may be linked with IPV during pregnancy were included in the final model. These factors Maternal factors include- sociodemographic characteristics; residence (rural/urban), maternal age (15–24, 25–34, and ≥ 35), maternal age at first marriage (<18/ > = 18 years), education level (no education, primary, secondary, and tertiary), obstetric and clinical factors; prior adverse birth outcomes (low birth weight, preterm birth, small-for-gestational age, stillbirth, or miscarriages- yes/no), parity (null, 1–3, 3+), birth interval (< 24, > = 24 months), any medical conditions like HIV/AIDS, psychiatric conditions (yes/no), decision on family planning use (wife only, husband only, or both), substance use during current pregnancy (cigarette, alcohol, or khat- yes/no), and antenatal care for this current pregnancy (yes/no), mid-upper arm circumference (MUAC), a commonly used method in the diagnosis of acute malnutrition in pregnant women, was employed for anthropometric assessment to assess the nutritional status of the women. Further the current husband or partner related factors such as substance use in the form of either cigarette, alcohol, or khat- (yes/no) were included in the final model.

#### Operational definition

*Emotional IPV*. Defined as mothers who experienced any of the following; have been insulted by your husband by using abusive language that made you feel bad, insulted in front of others, have been scared or intimidated you on purpose, or have been threatened by your husband with an object such as a stick, belt, knife, gun, etc. by a current husband/partner/boyfriend during the current pregnancy [3].

*Physical IPV*. Defined as mothers experiencing any of the followings; being slapped or having something thrown at her that could hurt her, being pushed or shoved, being hit with a fist or something else that could hurt, being kicked, dragged, being choked or burnt on purpose, and/or being threatened with/actually having, a gun, a knife, or another weapon used on her by a current intimate partner during the current pregnancy [3].

*Sexual IPV*. Defined as mothers experiencing any of the followings; being physically forced to have sexual intercourse when she did not want to, having sexual intercourse because she was afraid of what her partner might do, and/or being forced to do something sexual that she found humiliating or degrading to her by an intimate partner during the current pregnancy [3].

### Data collection and procedures

The IPV-related questions were adapted from the WHO 2005 Multi-Country Study to assess violence against women [3] and modified to suit the Ethiopian context, as per the women’s domestic violence assessment tool in the Ethiopian Demographic and Health Survey [32] women’s domestic violence assessment tool. The questionnaire consisted of sociodemographic characteristics, obstetric, medical, and behavioral, and experiences of IPV during the index pregnancy.

The questionnaires were prepared with thirteen IPV-related questions to assess the experience of the three dimensions of IPV (6- Physical, 4- Emotional, and 3- Sexual related questions) during the current pregnancy to determine the exposure to IPV.

Mid-upper arm circumference (MUAC), a commonly used method in the diagnosis of acute malnutrition in pregnant women, was employed for anthropometric assessment to assess the nutritional status of the women.

The MUAC of each woman, measuring at the midpoint from the shoulder to the elbow of the left arm, has been made using a unelastic and stretchable MUAC tape. Measurements were taken to the nearest 0.1 cm.

The data were collected by face-to-face interviews using a standardized, structured, and pretested questionnaire. Mothers were interviewed in separate rooms to ensure their privacy. The interview was done by trained midwives or nurses who were working at the ANC clinic with two separate shifts in each study hospital. The questionnaire was translated from English into the local language (Amharic) and back to the English version to keep its consistency. A pre-test was done on 5% of the sample size in non-selected public hospitals found in the Amhara region. However, we found no confusion during pre-testing and no amendment was done for IPV-related and other questions in the questionnaires. Further three days of training was provided for data collectors and supervisors.

### Data management and Bayesian statistical analysis approach

Data entry and coding were done using Epi-data version 3.1. Data cleaning and analysis were carried out using Stata version 17.

The Bayesian analysis approach is a strong statistical approach in health sciences research that considers data at hand and pre-existing data with the idea of the posterior distribution [33–36].

As opposed to the usual logistic regression, which treats the unknown parameters as fixed constants. When using Bayesian logistic regression, the parameters are viewed as random variables that could change depending on a prior probability distribution. This change can be interpreted as entirely stochastic for a data-driven model, but the Bayesian approach can also interpret it as a belief of uncertainty [37].

In a Bayesian formulation, a probability distribution can be used to illustrate the degree of uncertainty surrounding each parameter’s value. The likelihood function, which reflects information about the parameters contained in the data, and the prior distribution, which quantifies what is known about the parameters before observing data, are the main elements of a Bayesian analysis. The posterior distribution, which provides complete information about the parameters after the data have been observed, can be formed by simply combining the prior distribution and likelihood.

Markov Chain Monte Carlo (MCMC) simulation with Metropolis-Hastings sampling algorithm was carried out with 360599 total iterations. After 6000 burn-in terms were discarded, 900,000 samples were generated from the full posterior distribution. For the fixed effect and the gamma distribution with scale = 0.1 and shape = 0.1 for the variance of random effect, a noninformative normal prior distribution with mean = 0 and variance = 10^6^ was used. Convergence assessment plots were used to demonstrate the convergence of Markov chains before any inferences could be drawn from the posterior distribution.

Time series (history) plots, density plots, Autocorrelation plots, and Gelman-Rubin statistics were used to assess the convergence algorithm. Summary statistics were carried out from the posterior distribution. A Bayesian multivariable logistic regression model was performed to identify the predictors of IPV during pregnancy.

Finally, an adjusted odds ratio (AOR) with a 95% Bayesian credible interval was reported for statistically significant factors associated with IPV during pregnancy.

#### Ethics approval and consent to participate

Ethical approval for the study was obtained from the Research and Ethics Committee (REC) of Dream Science and Technology College, Dessie Campus, with an approval letter DSTC/0031/2021. Furthermore, this study, conducted in selected hospitals with permission letters from the respective health sectors (District health offices), adhered to the World Health Organization’s (WHO) standards for handling ethical issues in violence studies. Complete information on the study’s purpose, objectives, procedures, potential risks and benefits has been provided to the participants. Pregnant women were assured of strict confidentiality regarding any information obtained from them, aligning with WHO guidelines. Each participant was explicitly informed of their right to refuse participation, pose any unclear questions, and discontinue the interview at any point for any inconvenience. Finally, we obtained informed oral consent from each participant. In addition, the same consent was obtained from pregnant women less than 18 years of age because in Ethiopia, women are fully responsible and can make decisions regarding their family and themselves after they get married.

The confidentiality and privacy of the data were prioritized by avoiding personal identifiers and storing data securely on locked personal computers, in accordance with WHO standards for ethical conduct in violence research.

## Results

### Sociodemographic characteristics of participants

In this study, 402 participants were involved with a response rate of 96.2%. The mean age of the participants was 27.12 years ± 5.25 SD years. Of the total participants, 27% of women were married below the age of 18 years, 49.2% of women resided in rural areas, 37% of women have no education, and 50,8% of housewife women experienced any intimate partner violence during the current pregnancy **(Table 1)**.

**Table 1:** Sociodemographic characteristics participants and distributions of IPV during pregnancy in public hospitals, Amhara region, Ethiopia.

Predictor	Category	IPV during pregnancy	
		Yes (%)	No (%)
**Age (in years)**			
	15–24	42(33.3)	77(27.9)
	25–34	69 (54.8)	171 (62.0)
	35–49	15 (11.9)	28 (10.1)
**Age at first marriage (in years)**			
	Less than 18	34 (27.0)	68 (24.6)
	18 and above	92 (73.0)	208 (75.4)
**Residence**			
	Urban	64 (50.8)	172 (62.3)
	Rural	62 (49.2)	104 (37.7)
**Marital status**			
	Married	120 (95.2)	272 (98.6)
	Unmarried	6 (4.8)	4 (1.4)
**Religion**			
	Orthodox	73 (57.9)	134 (48.6)
	Muslim	48 (38.1)	141 (51.1)
	Others^++^	5 (4.0)	1 (0.4)
**Education level of mother**			
	No education	47 (37.3)	61 (22.1)
	Primary	31 (24.6)	110 (39.9)
	Secondary	26 (20.6)	61 (22.1)
	Tertiary	22 (17.5)	44 (15.9)
**Educational level of husband**			
	No education	36 (28.6)	51 (18.5)
	Primary	33 (26.2)	70 (25.4)
	Secondary	28 (22.2)	94 (34.1)
	Tertiary	29 (23.0)	61 (22.1)
**Occupation of mother**			
	Housewife	64 (50.8)	143 (51.8)
	Farmer	23 (18.3)	46 (16.7)
	Merchant	9 (7.1)	32 (11.6)
	Others^+^	30 (23.8)	55 (19.9)
**Family size**			
	< 5	85 (67.5)	195 (70.7)
	≥ 5	41 (32.5)	81 (29.3)
**Head of household**	Wife only	5 (4.0)	17 (6.2)
	Husband only	69 (54.8)	154 (55.8)
	Both	52 (41.3)	105 (38.0)

Key: **“*Others***^***+***^***”*** include student, daily labor, self-employed, and **“*Others***^***++***^***”*** protestant, Fete-Waka

### Obstetric and medical conditions of participants

In this study, 37% of pregnant women were multi-parity, 5.6% of pregnant women were seropositive, 25.4% of pregnant women had experienced medical problems during pregnancy and 23% of pregnant mothers had exposure to previous adverse birth outcomes (i.e., preterm birth, low birth weight, Stillbirth, and abortion) **(Table 2).**

**Table 2:** Obstetric and medical conditions of participants in public hospitals, Amhara region, Ethiopia.

Predictor	Category	IPV during pregnancy	
		Yes (%)	No (%)
Parity	Zero	61 (48.4)	135 (48.9)
	One to Two	18 (14.3)	59 (21.4)
	Three and above	47 (37.3)	82 (29.7)
Nutritional status of Mother (in MUAC)	SAM (<21cm)	5 (4.0)	5 (1.8)
	Moderate (21–22.9cm)	16 (12.7)	30 (10.9)
	Normal (≥ 23 cm)	105 (83.3)	241 (87.3)
Birth interval for this pregnancy			
	< 2 years	17 (26.2)	32 (22.7)
	≥ 2 years	48 (73.8)	109 (77.3)
Gestational Age at ANC initiation			
	<4 months	46 (39.7)	126 (47.0)
	≥ 4 months	70 (60.3)	142 (53.0)
HIV Status of the mother			
	Reactive	7 (5.6)	9 (3.3)
	Non-reactive	119 (94.4)	267 (96.7)
Medical problems during this pregnancy	Yes	32 (25.4)	41 (14.9)
	No	94 (74.6)	235 (85.1)
Previous Adverse birth outcomes (stillbirth, Preterm, LBW, abortion)	Yes	29 (23.0)	45 (16.3)
	No	97 (77.0)	231 (83.7)

### Behavioral-related characteristics of participants

Of the total participants who had experienced any IPV during pregnancy; 18.3% of pregnant women were chewing khat at least once per week during pregnancy, 52.4% were drinking alcohol-containing drinks at least once per week during pregnancy, and 58.7% of women had their husbands/partner drinking alcohol at least once per week before the study period **(Table 3)**.

**Table 3:** Behavioral-related characteristics of participants in public hospitals, Amhara region, Ethiopia.

Predictor variables	Category	IPV during pregnancy	
		Yes (%)	No (%)
Mother chewing khat during pregnancy			
	Yes	23 (18.3)	52 (18.8)
	No	103 (81.7)	224 (81.2)
Mother smoke cigarette during pregnancy			
	Yes	2 (1.6)	1 (0.4)
	No	124 (98.4)	275 (99.6)
Mothers drink Alcohol during pregnancy			
	Yes	66 (52.4)	112 (40.6)
	No	60 (47.6)	164 (59.4)
Husband/partner currently chewing khat			
	Yes	41 (32.5)	102 (37.0)
	No	85 (67.5)	174 (63.0)
Husband/partner currently smokes a cigarette			
	Yes	5 (4.0)	11 (4.0)
	No	121 (96.0)	265 (96.0)
Husband/partner currently drinks Alcohol			
	Yes	74 (58.7)	110 (39.9)
	No	52 (41.3)	166 (60.1)

### Prevalence of Intimate Partner Violence during pregnancy

The overall prevalence of experience of any Intimate Partner Violence (IPV) during pregnancy was 31.3% [95%CI, 26.98% - 36.07%]. Furthermore, 19.9%, 10.7%, and 18.4% of pregnant women had experienced emotional, physical, and sexual IPV during their pregnancy period, respectively. In addition, 7% of pregnant women had experienced all forms of IPV during the current pregnancy.

## Bayesian logistic regression analysis

In the final Bayesian multivariable logistic regression, residence site, age of women, women’s educational level, adverse birth outcome, chronic medical problem, husband substance use, decision maker of family planning, and the number of ANC visits are statistically significant variables.

After adjusting other variables women living in rural dwellers were two times more likely to experience IPV during pregnancy compared to women living in urban residences [AOR = 2.30: 95% CI 1.41, 3.72].

Study participants whose age was between 25–34 and whose age 35 and above years were 62% and 65% less likely to have IPV during pregnancy than study participants whose age was between 15–24 years [AOR = 0.38: 95% CI 0.18, 0.69], [AOR = 0.35: 95% CI 0.13,0.73] respectively.

This study indicated that women who had primary and secondary education 0.47 and 0.64 were less likely to experience IPV during pregnancy compared to those who had no formal education [AOR = 0.47: 95%CI 0.24, 0.81], [AOR = 0.64: 95%CI 0.41, 0.92] respectively.

The odds of IPV experience among pregnant women who had four or more ANC visits was 57% less compared to women who had less than four ANC visits [AOR = 0.43: 95% CI 0.25, 0.68].

Pregnant women who had no medical problems during/before pregnancy were 67% lesser odds of IPV experiences during pregnancy compared to those who had any medical problems AOR = 0.33: 95% CI 0.18, 0.54]. Similarly, pregnant women who had no adverse birth outcomes were 52% lesser odds of IPV experiences during pregnancy compared to those who had any adverse birth outcomes [AOR = 0.48: 95% CI 0.27, 0.80].

Household decisions made by husbands only had a 6 times higher risk of IPV exposure during pregnancy compared to those who decided by wives only [AOR = 6.45: 95% CI 3.01, 12.64]. Moreover, women whose husband uses substances had four times higher risk of IPV exposure compared to those who didn’t use substances [AOR = 4.33: 95% CI 1.68, 8.95] (**Table 4**).

**Table 4:** Factors associated with any IPV during pregnancy among pregnant women on ANC follow-up at selected public hospitals, Amhara region, Ethiopia.

Predictor	Category	SD	MCSE	AOR (95%CI)
Residence	Urban			
	Rural	0.583	0.086	2.30 (1.41 3.72) *
Age at first marriage	<18 years			
	> = 18 years	0.332	0.049	1.53 (0.98 2.31)
Age of women (in years)	15–24			
	25–34	0.129	0.017	0.38 (0.18 0.69) *
	35 and above	0.154	0.013	0.35 (0.13 0.73) *
Educational level of women	No education			
	Primary	0.143	0.020	0.47 (0.24 0.81) *
	Secondary	0.128	0.016	0.64 (0.41 0.92) *
	Tertiary	0.204	0.032	0.88 (0.55 1.31)
Previous adverse birth outcomes	Yes			
	No	0.135	0.023	0.48 (0.27 0.80) *
Chronic Medical problems	Yes			
	No	0.093	0.100	0.33 (0.18 0.54) *
Decision maker on family planning choices	Wife only			
	Husband only	2.496	0.133	6.45 (3.01 12.64) *
	Both	0.336	0.022	0.99 (0.49 1.75)
Substance use during pregnancy	Yes	0.331	0.048	0.79 (0.37 1.66)
	No			
Husband Substance use	Yes	1.922	0.281	4.33 (1.68 8.95) *
	No			
Parity	1–3			
	3 and above	0. 442	0.046	1.84 (0.11 2.88)
Birth interval	Less than 24			
	24 and above months	0.601	0.043	1.81 (0.93 3.22)
ANC Visits	Less than 4			
	4 and above	0. 110	0.016	0.43 (0.25 0.68) *

Note: *Adverse birth outcomes (preterm birth*, *low birth weight*, *& stillbirths) AOR-Adjusted Odds ratio*, *IPV-Intimate partner violence*

* statistically significant variables at 95% credible interval.

## Discussion

This study aimed to assess the prevalence and determinants of Intimate Violence among pregnant women at Public Hospitals in the Amhara region, Ethiopia. The prevalence of experience of any Intimate Partner Violence (IPV) during pregnancy is 31.3% [95%CI, 26.98% - 36.07%] which is higher than a study done in Kersa District, Oromia region Eastern Ethiopia (19.6%) [38], Amhara region, Northwest Ethiopia (25.4%) [5], Nigeria (5%) [39], the multilevel study conducted in Nigeria (15.2%) [40], Philippine (13%) [41], and South Africa (15%) [42].

However, the prevalence is lower than, in a study done in Awi Zone, Northwest Ethiopia (78%) [43], a study done in Gondar, Northwest Ethiopia (50.8%) [44], a study done in Abay Chomen District, Western Ethiopia (44.5%) [31], a study in Jimma, Ethiopia (81%) [45], the study conducted in Western Ethiopia (76.5%) [46] study done in Tanzania (33%) [47], the study conducted in Peru (52.2%) [48], a study done in southwest Nigeria (42.7%) [49], and study done in Eastern Saudi (39.3%) [50]. The discrepancy might be explained by the differences in the measurement and classification of IPV, sociocultural differences, and discrepancies in the strength of available global national, regional, and district-level legal legislation implemented to control violence against women.

The study indicated that women who had attended primary and secondary education were less likely to experience IPV during pregnancy compared to those who had no formal education.

This finding is in line with a study done in Abay Chomen District, Western Ethiopia [31], a study done in Egypt [11], a study done in southwest Nigeria [49], a multilevel study conducted in Nigeria [40], the study conducted in Western Ethiopia [46], the study done in Swaziland [51], and the study done in Zambia [52]. This could be justified by when the education level of women increased their awareness towards legal legislation to protect their rights, available health services, and refusal to harmful societal norms.

The likelihood of IPV among pregnant women who had any medical problems during/before pregnancy was three-fold higher compared to those who did not have any medical problems. This could be justified by women facing medical problems during pregnancy being exposed to medical expenses, absenteeism from work, the difficulty of caring for their family, and so on. Therefore, they are more likely to be violated compared to those women who had no medical problems.

Decisions on household issues made by husbands only had 6 times higher risk of IPV exposure during pregnancy compared to those who decided by wives only. This finding is supported by the study done in Awi Zone, North Western Ethiopia [43]. This could be explained by women living in households dominated by husbands/partners’ decision-making roles are prone to different sociocultural, socioeconomic, and access to health care services.

The odds of IPV experience among pregnant women who had four or more ANC visits was 57% less compared to women who had less than four ANC visits. This finding is comparable with other studies carried out in South Africa [53], Gondar [5], and Debre Markos town [54]. This may be because having an ANC visit increases the likelihood that the woman will get information on sexual and reproductive health, including violence, from healthcare professionals.

Women whose husband uses substances had a four times higher risk of IPV exposure compared to those who didn’t use substances. This finding is similar to the study done in Awi Zone, Northwestern Ethiopia [43], the study done in Gondar, Northwest Ethiopia [44], the study done in Egypt [11], the study done in Nigeria [39], the study done in Philippine [41], a study done in Eastern Saudi [50], the study conducted in Western Ethiopia [46], the study done in Eastern Sudan [55] and a systematic review done on African studies [56]. This may be because using drugs or alcohol can lead to anger and impair judgment, which raises the chance of violence. Furthermore, some people may purposefully drink to conceal their antisocial behavior, such as violence toward their spouses, behind the booze [57].

The study indicated that being of older age significantly decreases the likelihood to have IPV during pregnancy. This is supported by the findings of the study conducted in eastern Ethiopia [58]. This could be due to older women having more access to information about their legal rights.

## Strength and limitation of the study

The main strength of this study is that the use of validated instruments from a WHO multi-country study on violence against women. As a limitation, first, due to social desirability bias, some respondents may not volunteer to disclose their use of violence because it is a sensitive subject, which results in underreporting. Second, no qualitative methodologies were added to the study. Finally, the study might potentially share the drawbacks of cross-sectional study design.

## Conclusion

Based on the finding of the study, Intimate partner violence during pregnancy is a public health problem in the study area. One in three pregnant women experienced any type of violence during pregnancy. In the final model, residence site, age of women, women’s educational level, adverse birth outcome, chronic medical problem, husband substance use, decision maker on household issues, and the number of ANC visits are statistically significant independent variables of IPV among pregnant women. Therefore, preventive and control strategies against women’s violence during pregnancy should be developed at regional, zonal, and district levels.

We recommend including all types of IPV in the ANC services’ routine health assessment package and regularly training and educating health service providers about IPV against expectant women who attend ANC services. In addition, Community health professionals need to be empowered and supported adequately since they play a critical role in spreading awareness of the risks of IPV, especially for women residing in rural areas and with low educational levels. Policies aimed at improving ANC service attendance should be strengthened. Moreover, community-based study designs supplemented with qualitative studies are recommended.

## Supporting information

S1 File (DOCX)
