# Supplementary material for: Intimate Partner Violence and its associated factors among pregnant women receiving antenatal care. A Bayesian analysis approach
Source: PLoS One. 2024 Jul 11;19(7):e0304498. doi: 10.1371/journal.pone.0304498 (PMC11239075; doi:10.1371/journal.pone.0304498)
Supplement: S1 File — (DOCX) [file pone.0304498.s001.docx]

**Annex 1: Convergence assessment plots for significant parameters**


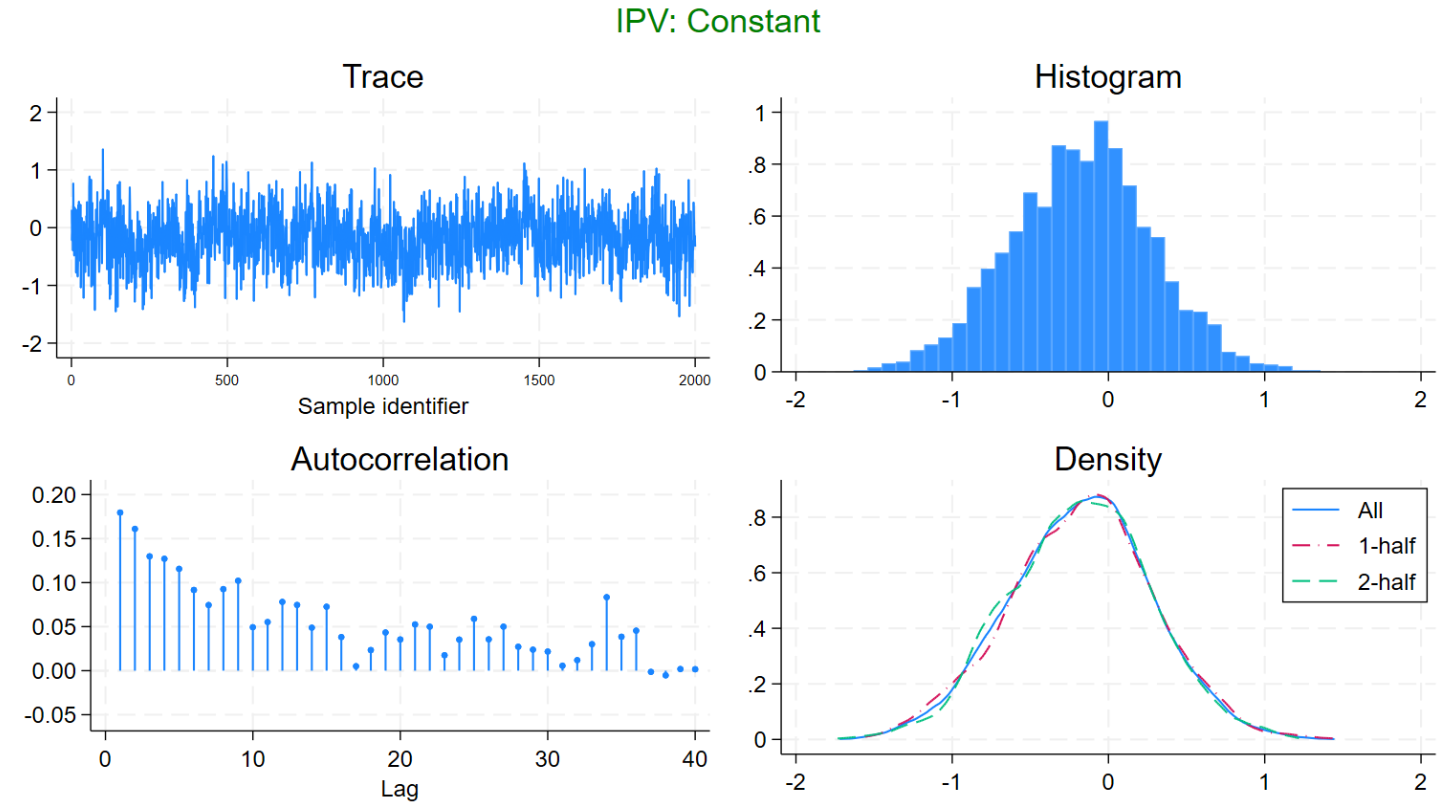


Figure 1. Empty model(constant)


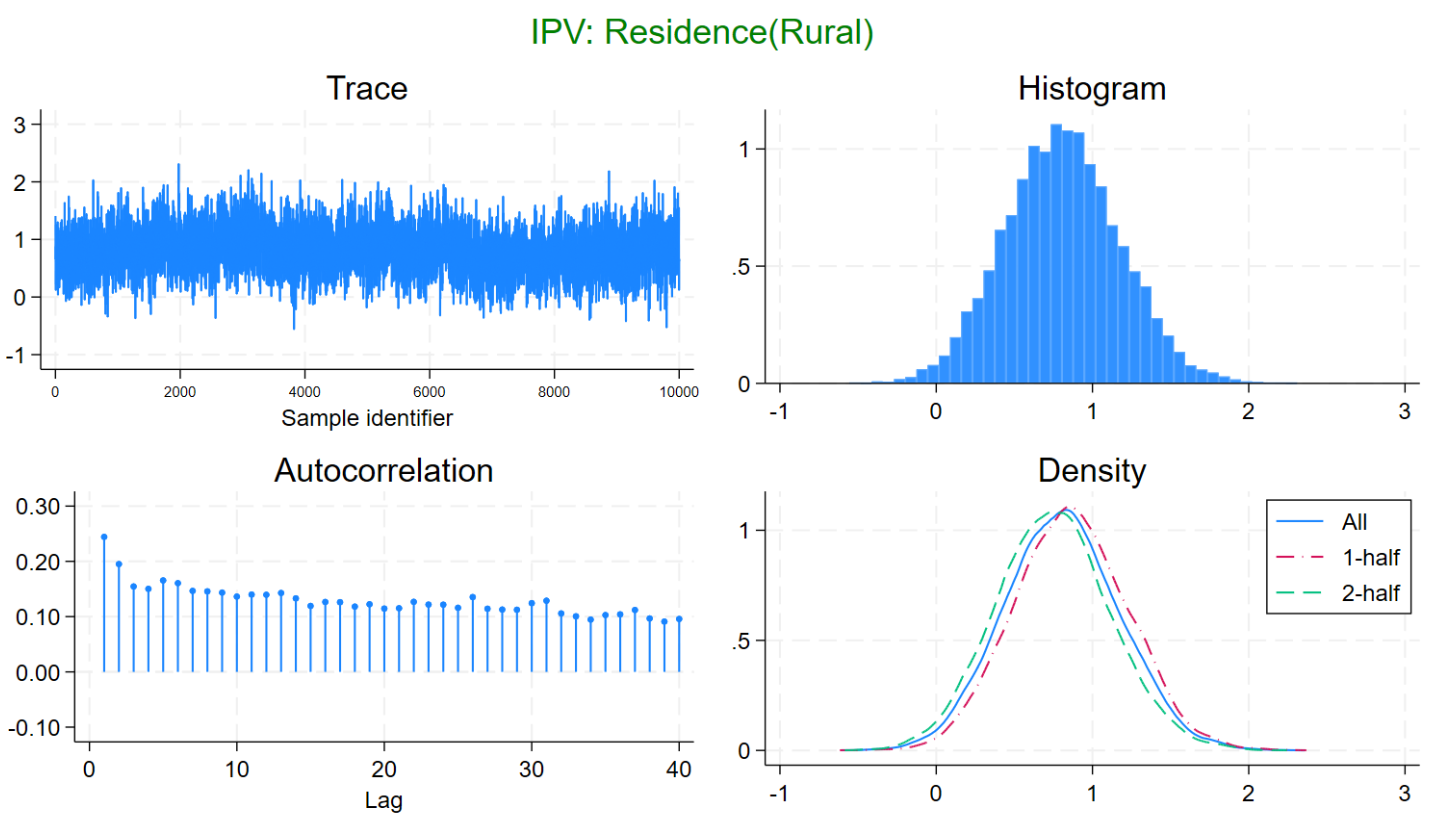


Figure 2. Residence site (Rural)


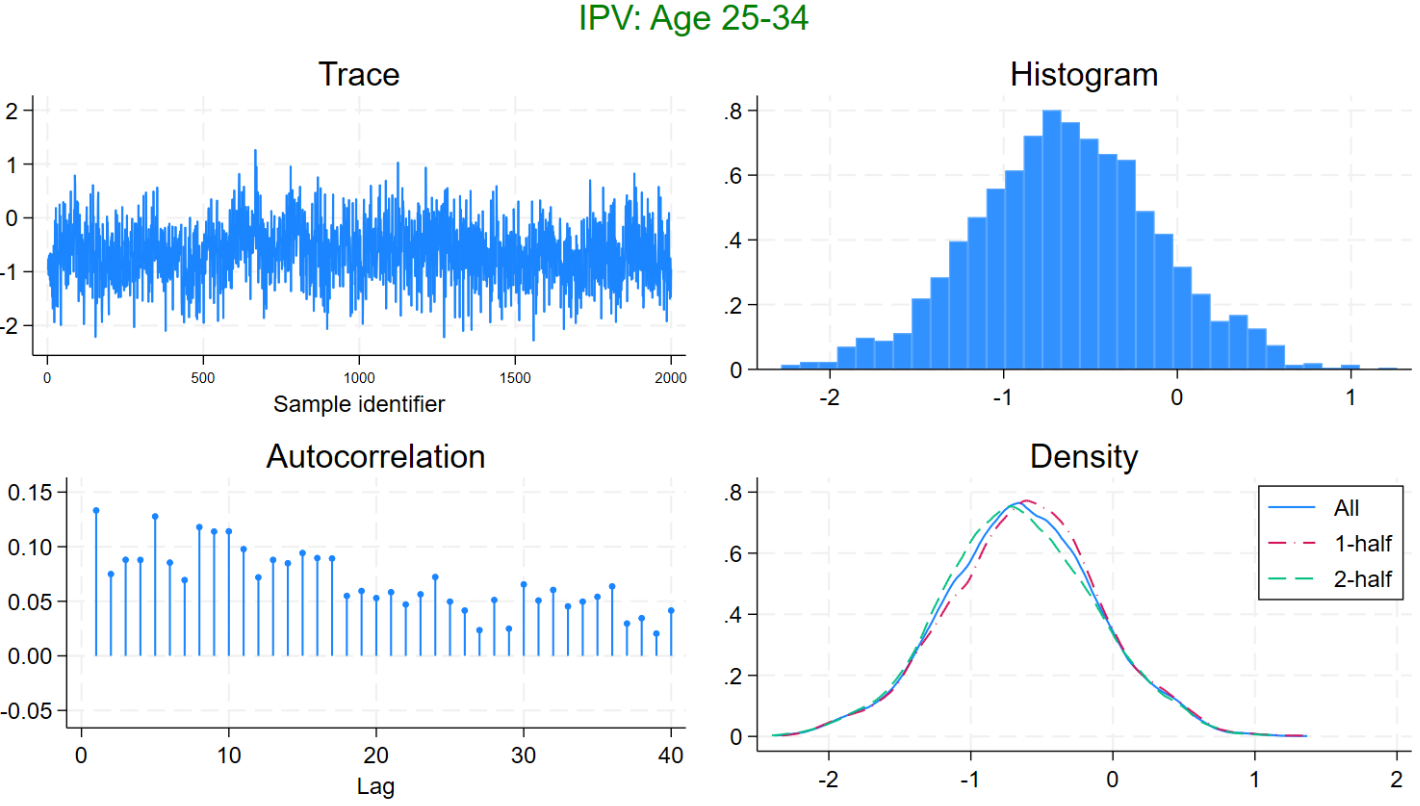


Figure 3. Woman aged 25-34


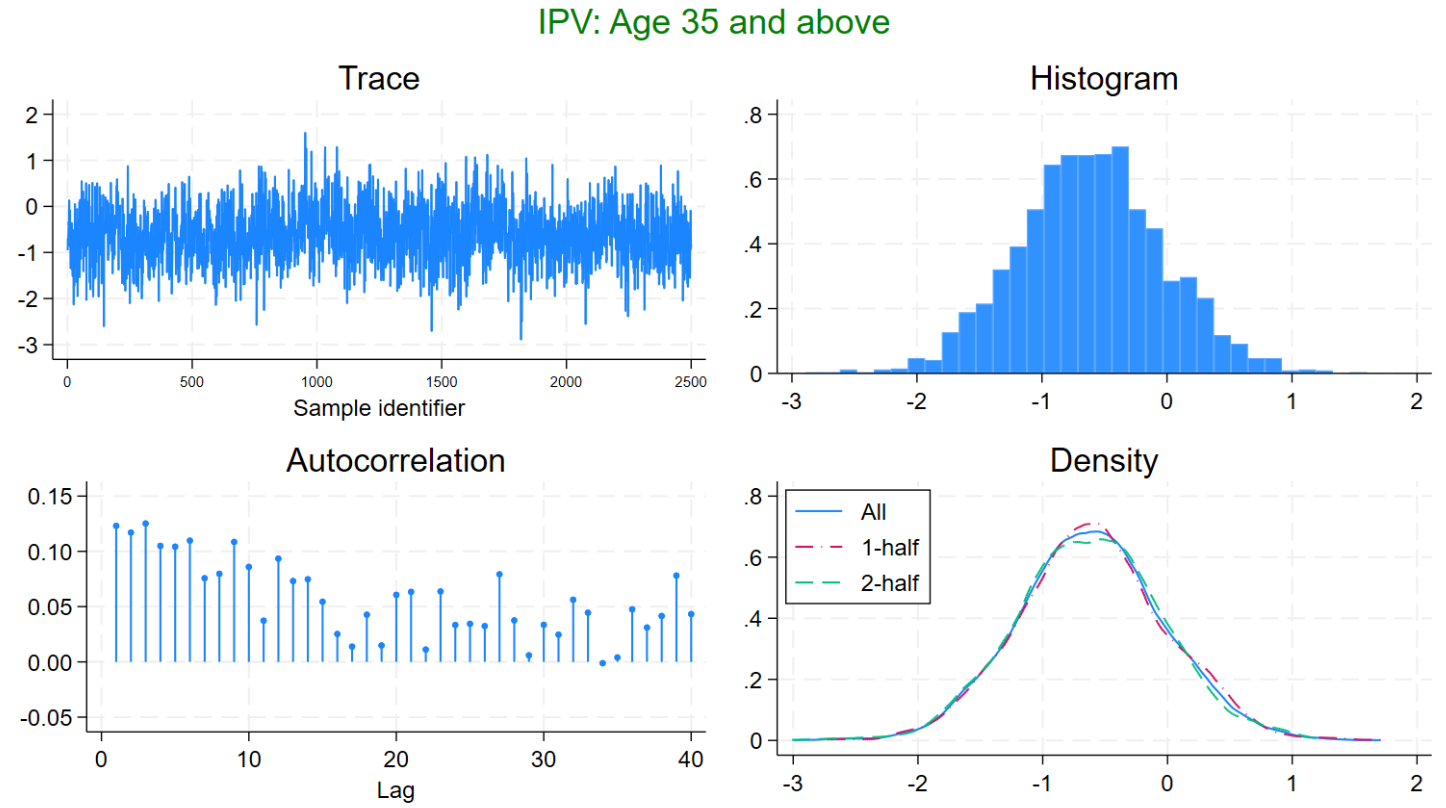


Figure 4. Woman aged 35 and above.


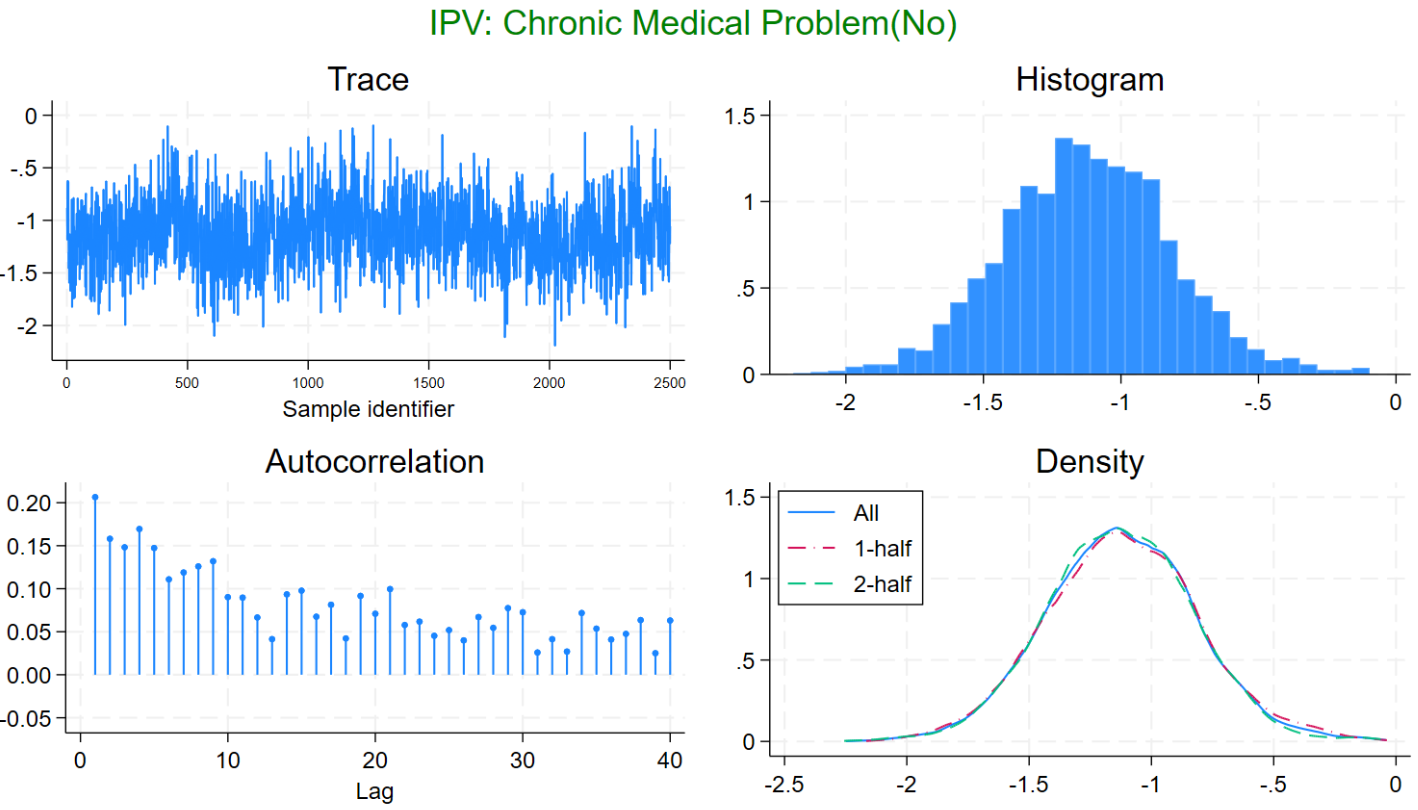


Figure 5. Woman with no medical problem


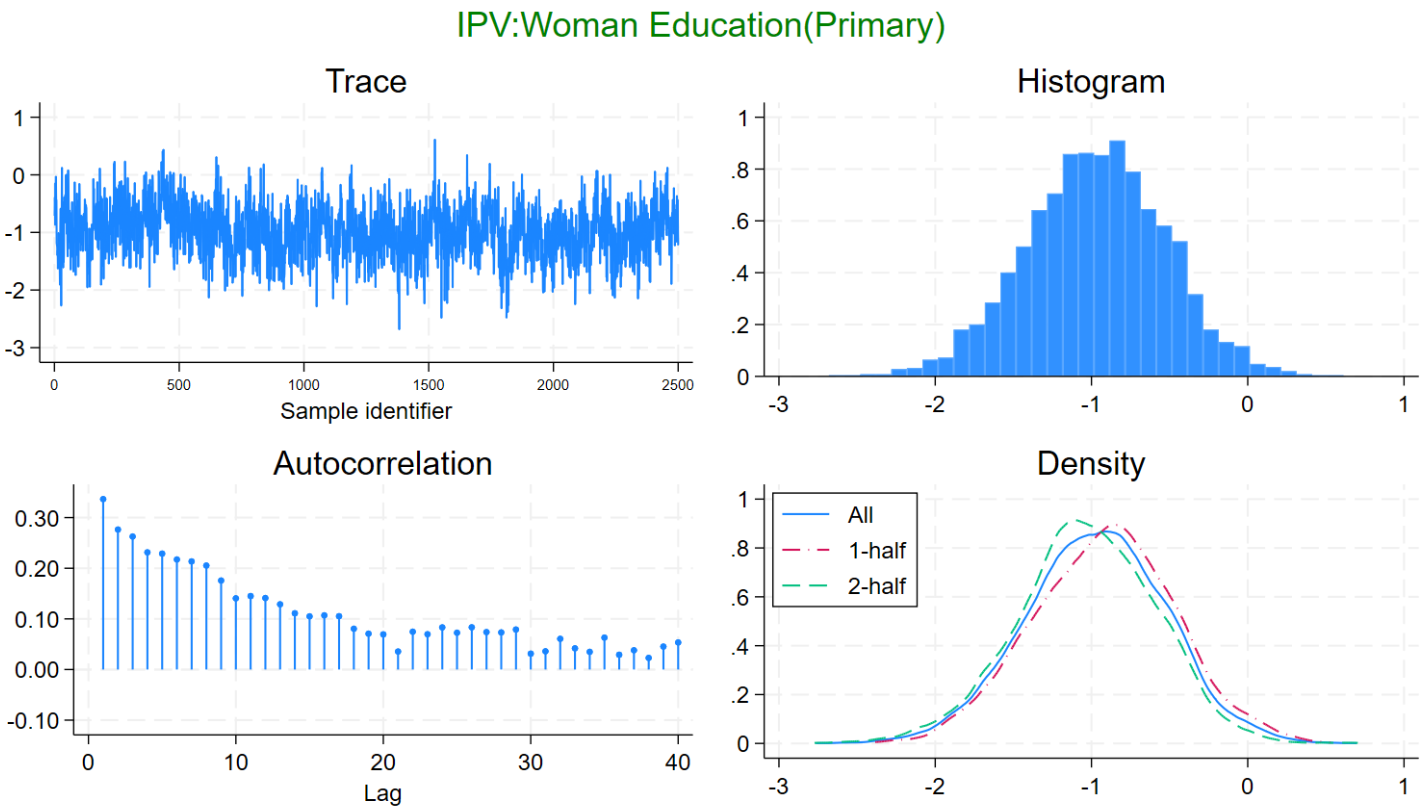


Figure 6. Woman education level (primary)


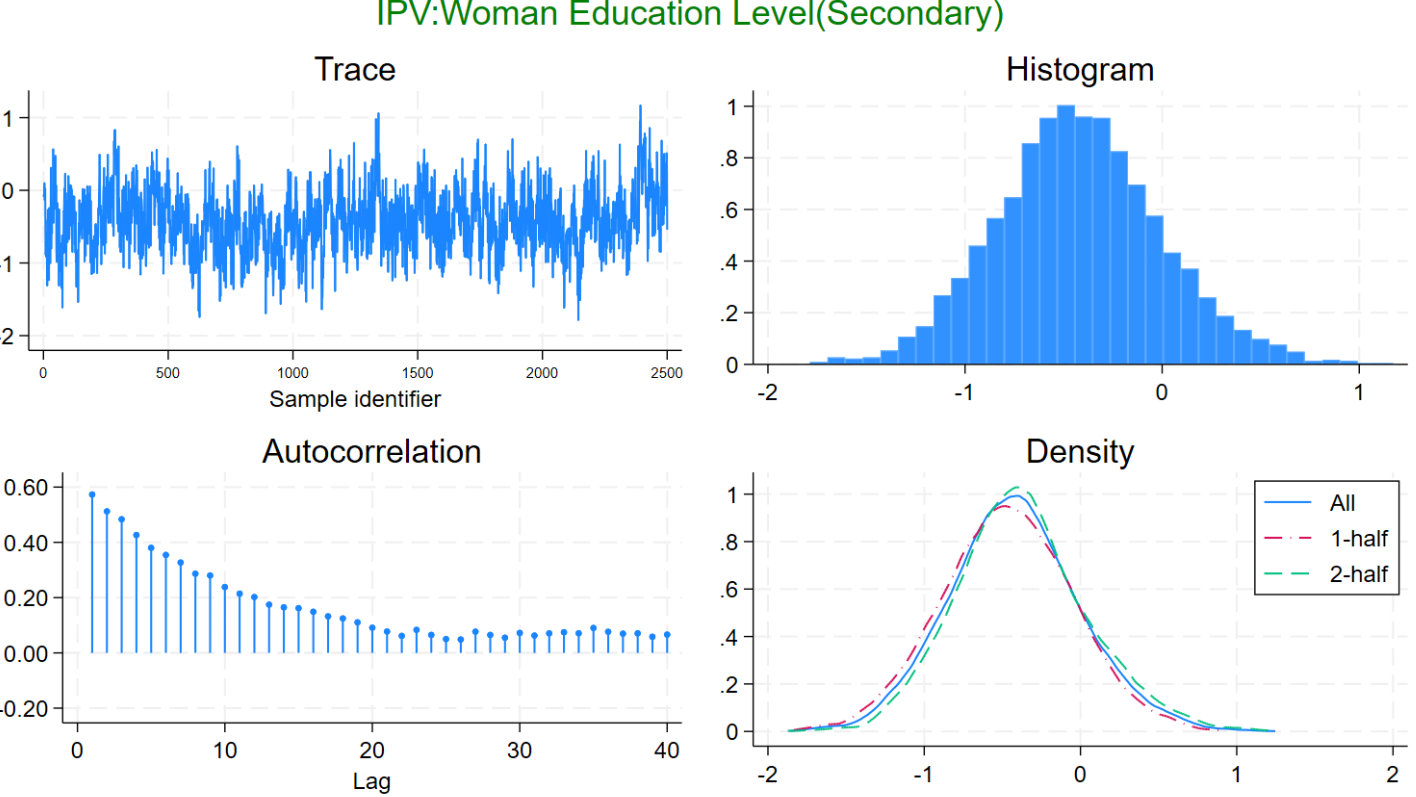


Figure 7. Woman education level (secondary)


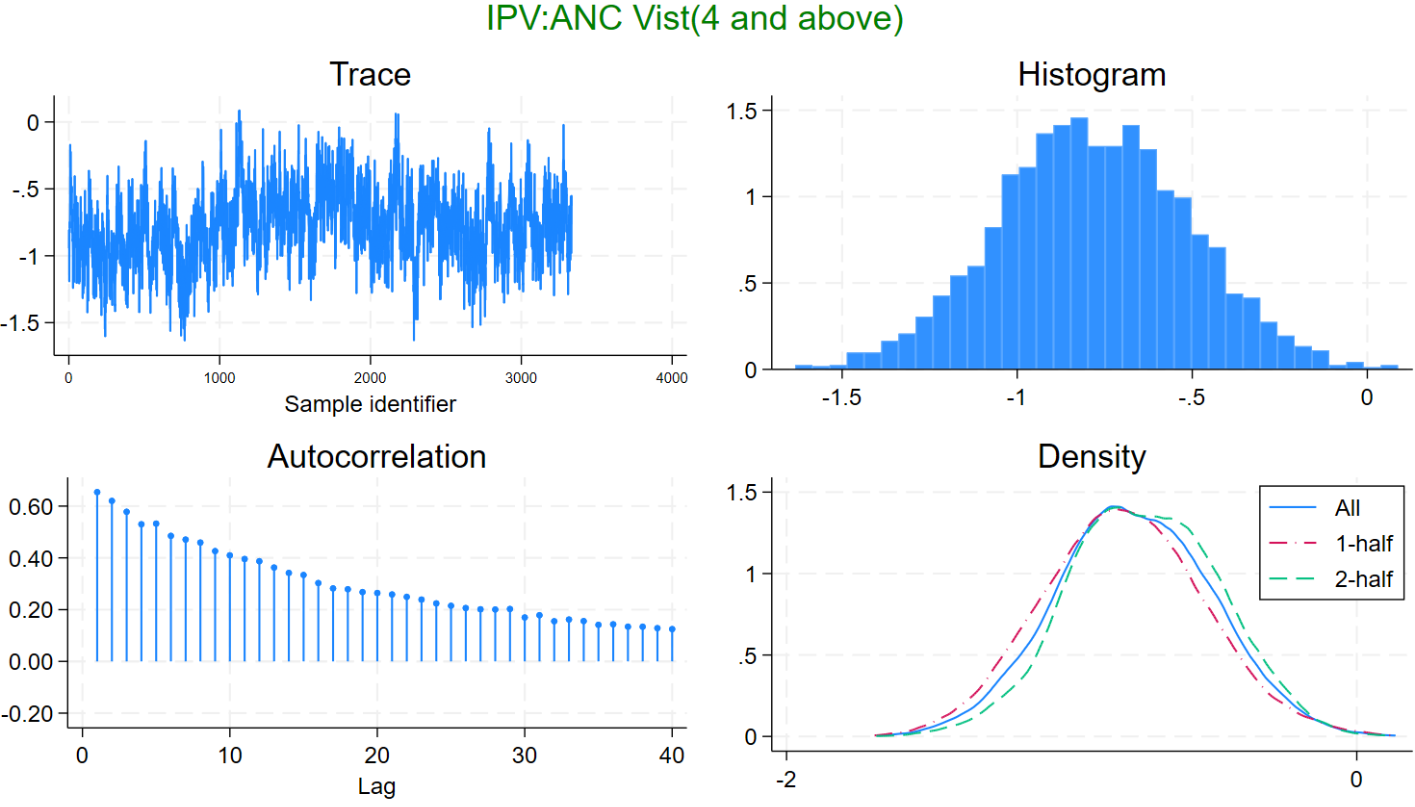


Figure 8. ANC visits 4 and above


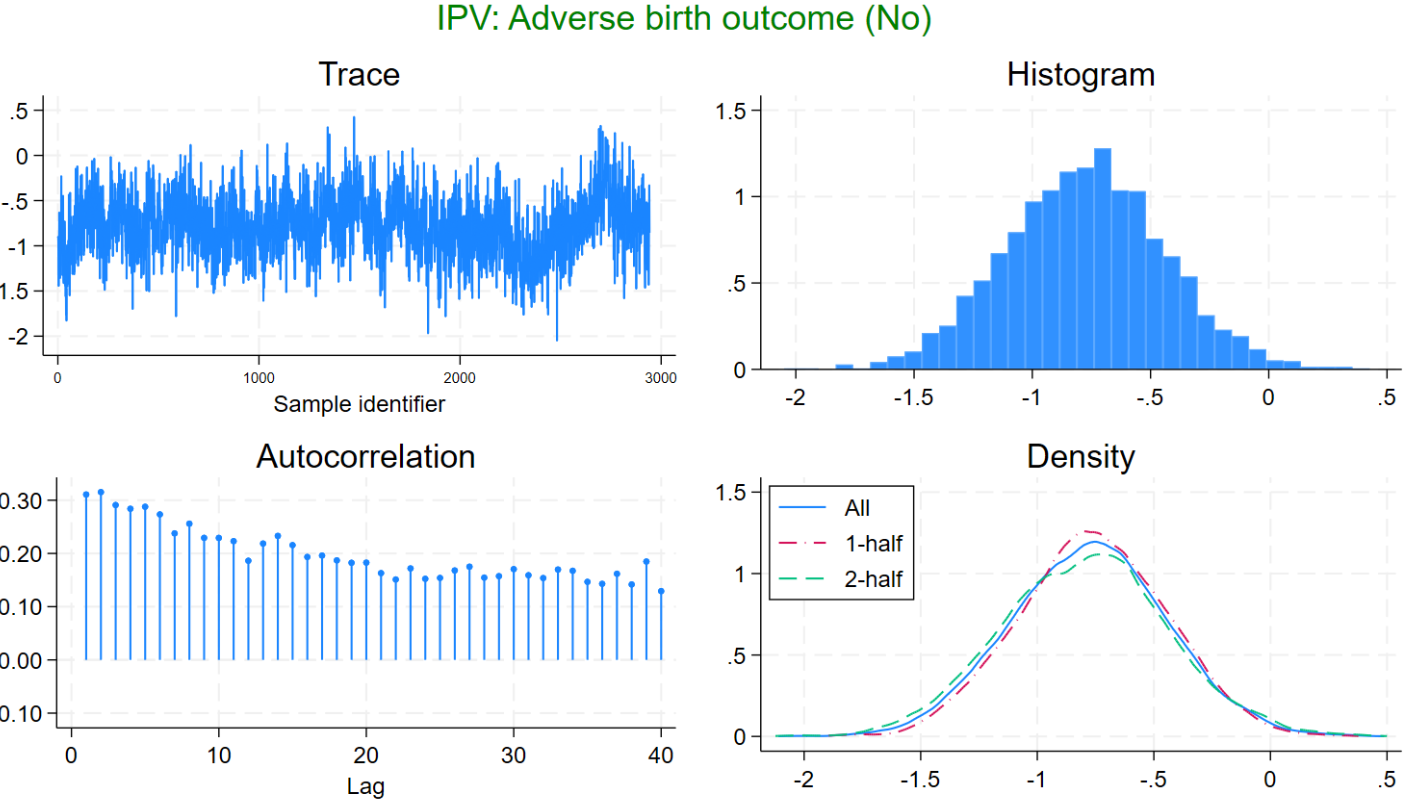


Figure 9. Woman who has adverse birth outcome


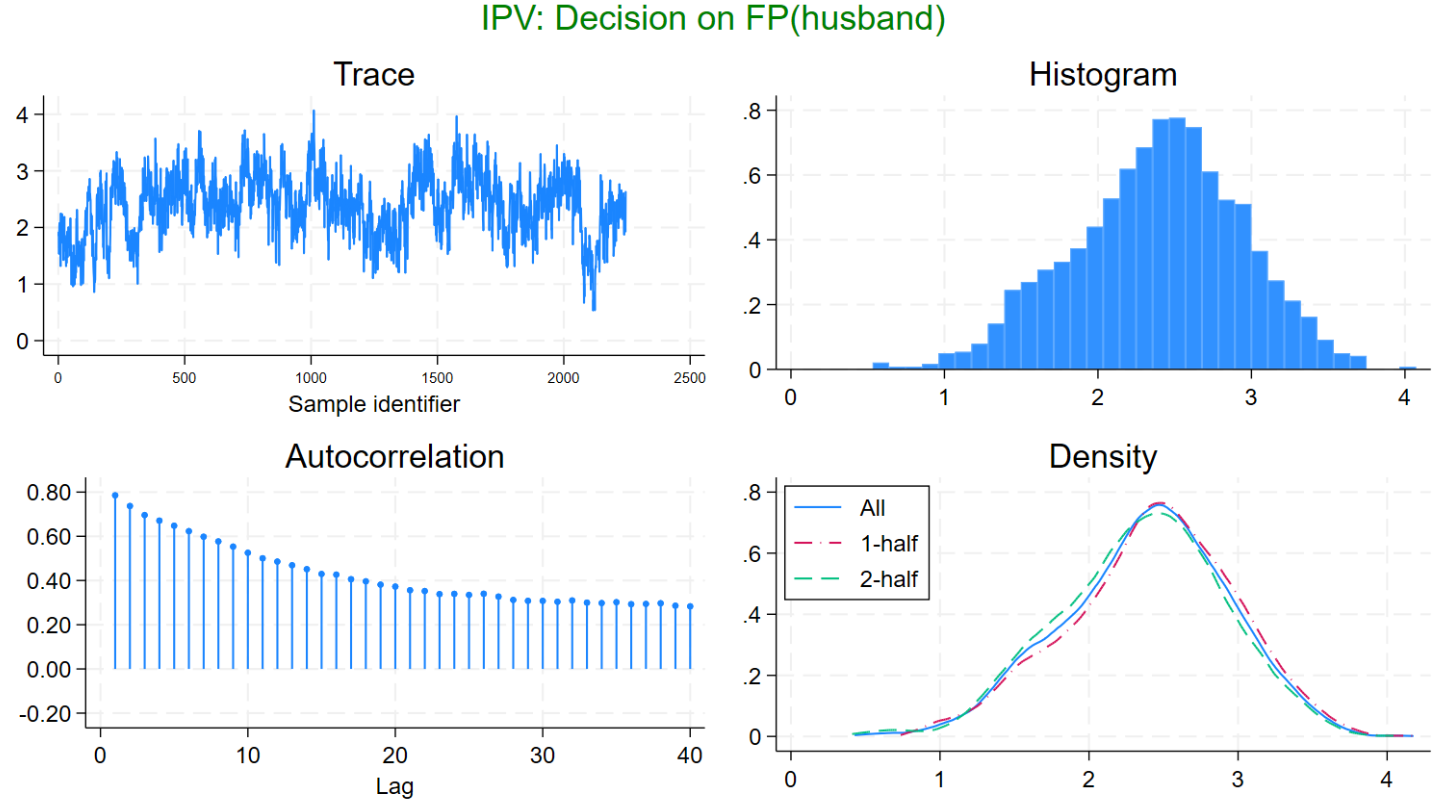


Figure 10. Decision on family planning made by husband only.


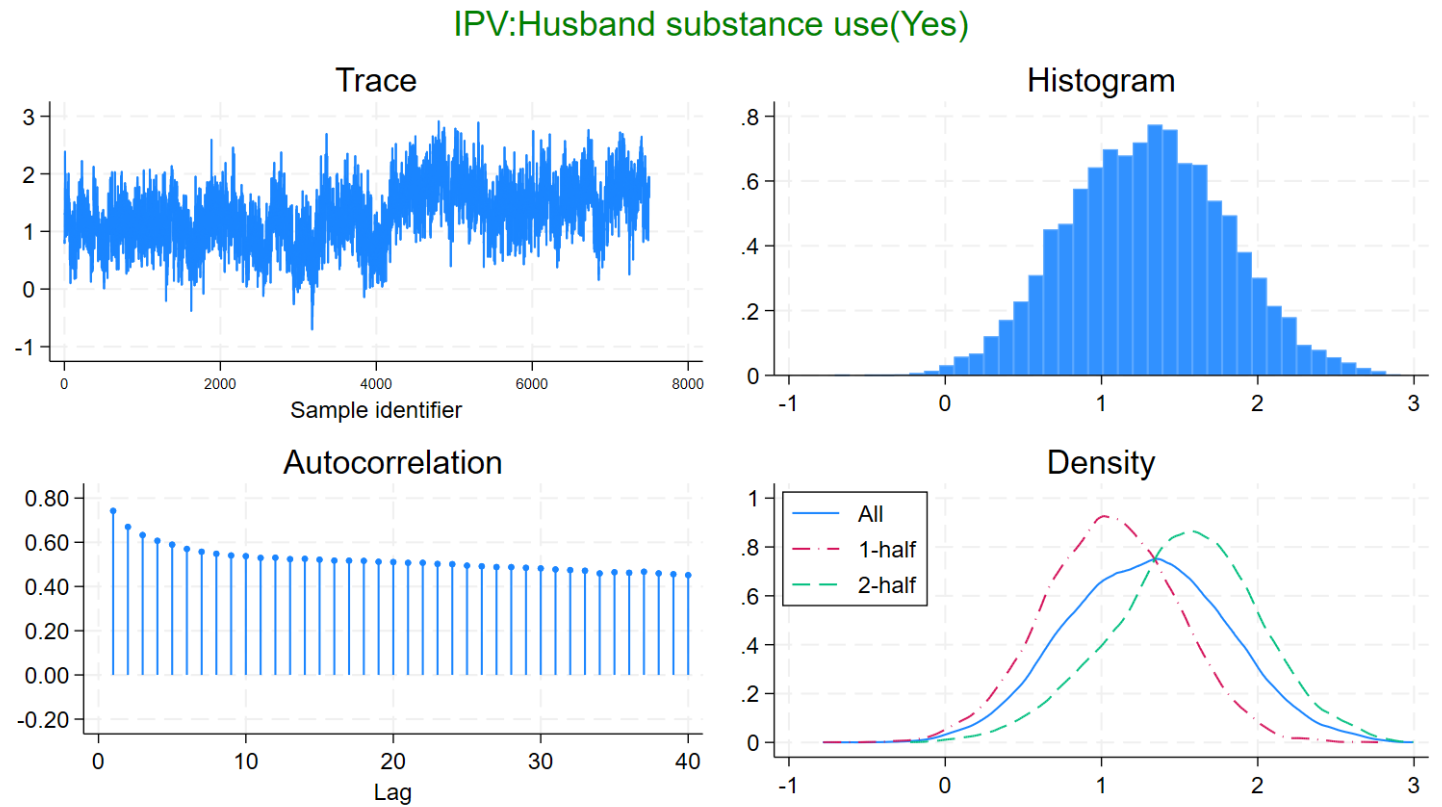


Figure 10. Husband substance use
